# Comparing computer-generated and pathologist-generated tumour segmentations for immunohistochemical scoring of breast tissue microarrays

**DOI:** 10.1038/bjc.2015.309

**Published:** 2015-09-08

**Authors:** Shazia Akbar, Lee B Jordan, Colin A Purdie, Alastair M Thompson, Stephen J McKenna

**Affiliations:** 1 School of Computing, University of Dundee, Dundee DD1 4HN, UK; 2 NHS Tayside Department of Pathology, Ninewells Hospital, Dundee DD1 9SY, UK; 3 Department of Surgical Oncology, MD Anderson Cancer Center, 1400 Pressler Street, Houston, Texas 77030, USA

**Keywords:** breast cancer, immunohistochemical assessment, digital pathology, tissue microarray, oestrogen receptor, Allred score, quickscore, image analysis software

## Abstract

**Background:**

Tissue microarrays (TMAs) have become a valuable resource for biomarker expression in translational research. Immunohistochemical (IHC) assessment of TMAs is the principal method for analysing large numbers of patient samples, but manual IHC assessment of TMAs remains a challenging and laborious task. With advances in image analysis, computer-generated analyses of TMAs have the potential to lessen the burden of expert pathologist review.

**Methods:**

In current commercial software computerised oestrogen receptor (ER) scoring relies on tumour localisation in the form of hand-drawn annotations. In this study, tumour localisation for ER scoring was evaluated comparing computer-generated segmentation masks with those of two specialist breast pathologists. Automatically and manually obtained segmentation masks were used to obtain IHC scores for thirty-two ER-stained invasive breast cancer TMA samples using FDA-approved IHC scoring software.

**Results:**

Although pixel-level comparisons showed lower agreement between automated and manual segmentation masks (*κ*=0.81) than between pathologists' masks (*κ*=0.91), this had little impact on computed IHC scores (Allred; 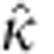
=0.91, Quickscore; 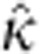
=0.92).

**Conclusions:**

The proposed automated system provides consistent measurements thus ensuring standardisation, and shows promise for increasing IHC analysis of nuclear staining in TMAs from large clinical trials.

With the improvements in clinical outcome in women treated for breast cancer such that 5 year survival now approaches 90% and 10 year survival 80%, adjuvant clinical trials require very large numbers of patients and tissue samples for biomarker studies to drive changes in clinical practice. Many such large clinical trials have moved towards generating tissue microarrays (TMAs; Ilyas *et al*, 2013) in an attempt to speed up the process of identifying and analysing biomarkers for which patients will, or will not, benefit from a particular therapeutic strategy. Such TMAs may contain multiple cores of tissue from each tumour measuring 0.6–1.0 mm in diameter with a mix of tumour, normal and other tissues incorporated in the core.

Although immunohistochemical (IHC) staining or molecular analyses of a single TMA slide with representation from 20–40 patients and multiple samples per patient is an efficient use of human tissue, antibodies and laboratory processes, there remains the problem of skilled detection and assessment of biomarkers. Such reading of the biomarker status is laborious and time consuming, requiring expert assessment. Thus, there has been increasing attention paid to the potential for automated reading of breast TMA biomarker slides rather than relying on pathology review (Madabhushi, 2009). However, accepting that expert specialist breast pathologist review is the ‘gold standard' for interpretation of biomarkers such as oestrogen receptor (ER) in breast cancer, any automated approach needs to be comparable to and consistent with such expert assessment.

In previous studies comparing automated and manual IHC scores (Arihiro *et al*, 2007; Fasanella *et al*, 2011; Rizzardi *et al*, 2012), evaluations were performed based on measurements retrieved after cell analysis. However the bottleneck in current image analysis algorithms lies in distinguishing healthy from cancerous tissue (Gurcan *et al*, 2009). In the digital pathology pipeline, tumour localisation is essential to focus scoring of cellular proteins to particular regions of interest. It is typically performed as a pre-processing step whereby the operator traces tumour regions manually. However advancements in image analysis in the last decade introduce prospects of using machines to automatically locate tumour in images of tissue with little or no human intervention. In this study we show the benefits of using such a system. The system adopted here has been designed to operate on a range of nuclear, cytoplasmic and membrane biomarkers. We use a nuclear IHC stain, ER, as an exemplar, and report comparisons between specialist breast pathologists and computational approaches for IHC assessment.

ER was selected as an exemplar as it is the most common target for IHC in breast cancer, provides clear nuclear staining with antibodies in clinical use, and is the basis for IHC scoring with clinical and research utility. ER thus presents a good exemplar for testing the methodological approaches used here.

In this study, manual segmentations replicated the current manner with which pathologists interacted with a widely used FDA-approved IHC scoring algorithm. Specifically, IHC ER Allred scores (Allred *et al*, 1990) and Quickscores (Detre *et al*, 1995) from computer-generated and manually obtained segmentation masks were compared. Here we report the development and evaluation of such software and the potential generalisability of the approach.

## Materials and methods

### Tissue microarrays

Breast TMAs were generated, for research purposes, from primary, previously untreated breast cancers using excess tissues from routine clinical practice after written, informed consent from the donor patients. Ethical permission was granted by Tayside Tissue Bank, Dundee, UK, under delegated authority of the Tayside Local Research Ethics Committee. In brief, surgically resected primary breast cancer from otherwise unselected patients was fixed in buffered formalin, stored at controlled temperature (18–22 °C) overnight and processed to formalin-fixed paraffin-embedded blocks. Whole mount sections stained with Haematoxylin and Eosin were marked to highlight relevant invasive cancer or normal tissue to allow TMA generation of up to six 0.6 mm cores per cancer. TMAs were then constructed using a manual tissue arrayer (Beecher Instruments Inc., Sun Prairie, WI, USA). Four micron TMA sections were cut, mounted onto poly-L-lysine-coated glass slides and subjected to staining for ER alpha localisation (6F11, 1 : 200; Novocastra Laboratories Ltd). Stained slides were scanned with lossy compression using an Aperio Scanscope XT (Aperio Technologies, Vista, CA, USA) on a × 20 objective with the optical doubler in place (equivalent to × 40 optical objective). Each slide was then segmented into the individual constituent stained spots, each spot representing a section from a tissue core.

Thirty-two uncompressed TIFF format images of TMA spots from thirty-two breast cancers were used. The perimeter of each spot was delineated and pixels exterior to this perimeter were excluded from subsequent analyses. Each spot image was ∼3000 pixels in diameter and contained invasive cancer.

### Manual segmentation of tumour regions

Tumour regions in the TMA spots were manually segmented using Aperio Technologies Spectrum Software with TMA Lab and the Webscope interface (Aperio Technologies). Segmentation involved manually tracing the boundaries of invasive tumour regions on a Wacom Bamboo Fun tablet (model CTH-461) using the stylus for precision; the software tool displayed filled regions overlaid on the TMA spot images as they were annotated. Each spot was annotated independently by two specialist breast pathologists (pathologist A and pathologist B), resulting in two sets of tumour masks. Pixels in each mask were labelled as either tumour (*T*) or non-tumour (*N*). Each spot took on average 23 min to annotate by each pathologist; however the task of annotating TMAs was spread over several days.

### Automated segmentation of tumour regions

Software implementing an image analysis algorithm was used to segment tumour regions automatically using an algorithm outlined in Supplementary Material 1. This algorithm uses clustering to group pixels into compact regions called superpixels such that boundaries of cellular compartments tend to lie on superpixel boundaries. A machine learning method was then used to label each superpixel as either tumour or non-tumour based on its colour, shape and visual texture, as well as similar properties from nearby superpixels. The algorithm was trained using a set of manually obtained segmentations.

Each of the thirty-two spots in the data set was automatically segmented twice, once using the algorithm trained on pathologist A's manual segmentation masks and once using the algorithm trained on pathologist B's manual segmentation masks. An 8-fold cross-validation experimental design was used in each case. In each fold, training took on average 30 min on an Intel Core i7-2600K processor; optimisation of this code can potentially reduce training time further.

### Comparing spot segmentations

**Figure 1:**
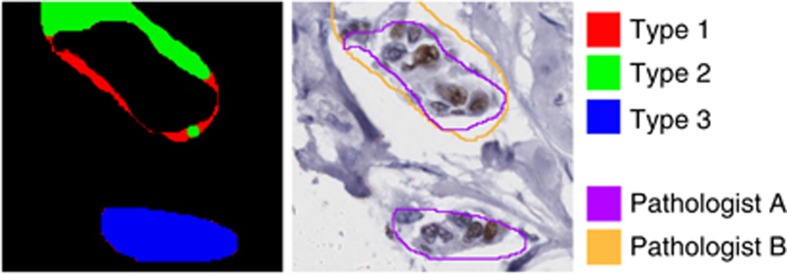
**Examples of Type 1 (red), Type 2 (green) and Type 3 (blue) disagreements.** Annotations drawn by pathologist A (purple) and pathologist B (orange) are shown on the right overlaid on the original image.

Each spot's two manual segmentation masks were compared with each other and with the computer-generated segmentation masks, by comparing the labels (*T* or *N*) assigned to each pixel. There are four possibilities when comparing a pixel's labels in two masks: (*T*, *T*), (*T*, *N*), (*N*, *T*) and (*N*, *N*). However, there are qualitative differences between segmented regions that are not well captured by simply counting the numbers of pixels that fall into each of these four categories. Therefore, when comparing two segmentation masks, we categorised pixel label disagreements into three types as follows (Figure 1, see Supplementary Material 2 for code).

Type 1: region boundaries in two segmentation masks are often separated along part of their lengths by distances of only a few pixels. Such discrepancies may arise from a lack of precision when using the stylus and/or from the lack of any clear visual boundary to annotate in the image. As such they are likely to be inconsequential for subsequent tasks such as IHC scoring because such small separations do not allow for the inclusion or exclusion of entire cells.

Type 2: disagreements which are not of Type 1 are large enough to encompass epithelial cells (Figure 1). A pixel disagreement is labelled Type 2, if it is not of Type 1 and it is in a region labelled as *T* in one mask that overlaps with a region labelled as *T* in the other mask. Type 2 disagreements can arise from differences of opinion about the spatial extent of a tumour region.

Type 3: Disagreements that are neither Type 1 nor Type 2 are designated Type 3, reflecting differences of opinion about whether or not a group of cells is malignant.

**Figure 2:**
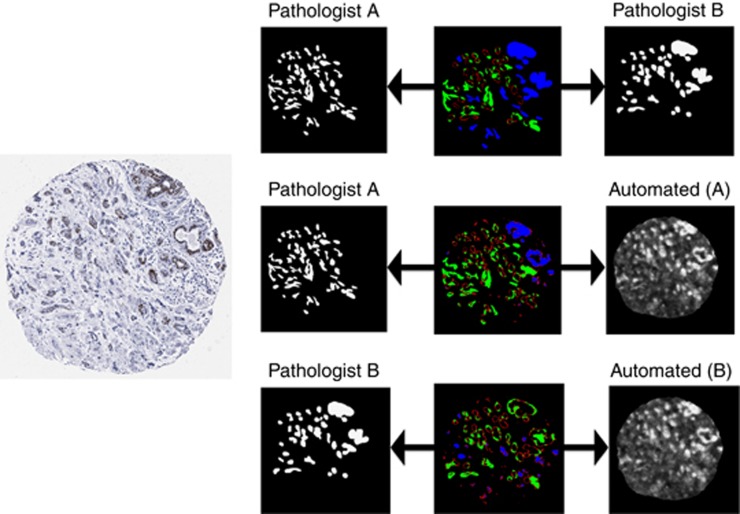
**A TMA spot (left) and colour-coded images showing types of disagreement between the two pathologists' manual annotations (top row), pathologist A and the algorithm trained by that pathologist (middle row), and pathologist B and the algorithm trained by that pathologist (bottom row).**

Disagreement types were visualised by computing difference images from pairs of segmentation masks and then colour-coding pixels for which the segmentations differed as Type 1, Type 2 or Type 3 (Figure 2).

### Oestrogen receptor scoring of segmented spots

The FDA-approved Aperio IHC Nuclear Version 10 algorithm (Aperio Technologies) was used to estimate ER scores based on the segmentation masks obtained. Only regions labelled as tumour (*T*) were passed to the scoring algorithm. The Aperio IHC algorithm identifies nuclei automatically and outputs a staining intensity score (ranging from 0 to 3) and an estimate of the percentage of positively stained cells. From these measurements, IHC scores (Allred score and Quickscore) were computed for manually and automatically obtained segmentation masks. Comparisons are reported to assess the extent to which differences in these segmentations affected scoring.

## Results

### Segmentation comparison

In pixel-level comparison of manually hand-drawn segmentation masks, pathologists differed in their labelling of 9% of pixels (Table 1). Automated segmentation masks were produced by training on either pathologist A or pathologist B. Comparisons of each pathologist's manual segmentations with those produced automatically revealed disagreements in 19% of pixels. Disagreements were similar between false positives (*N*, *T*) and false negatives (*T*, *N*).

**Figure 3:**
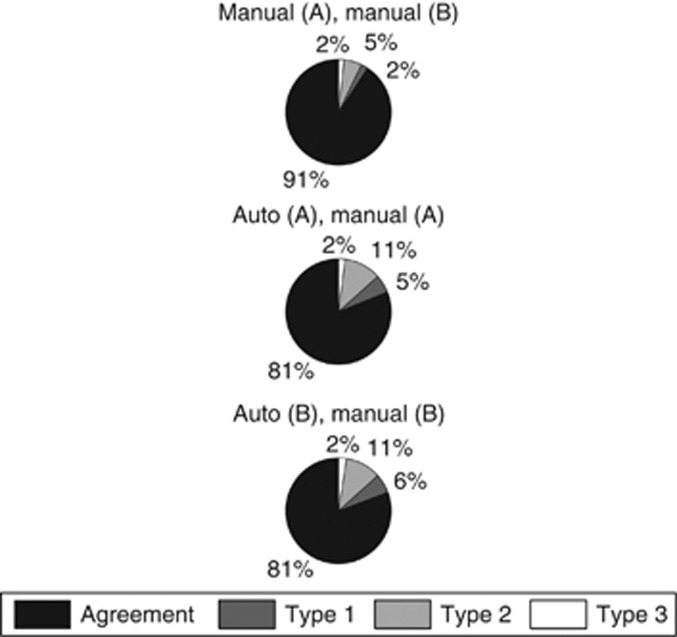
**Pie charts showing distribution of agreements, and Type 1, Type 2 and Type 3 disagreements between manual and automated segmentations.**

When distributions of agreements and disagreements were visualised (Figure 3), some variations were observed (Table 2). Of those pixels that were labelled differently by the two pathologists, 23% of disagreements were of Type 1, hence likely to have no impact on the ER score. The proportions of disagreements that were Type 1 when comparing automatic with manual segmentations were slightly higher (27%). The distributions of disagreements across remaining types (Type 2 and Type 3) were broadly similar whether comparing manual with automatic segmentation, or manual with manual segmentation. Although the average proportion of Type 3 disagreements was higher between manual segmentations (18±23%) compared with automated segmentations (11±12%), the s.d. was sufficient to indicate large variations between TMA spot assessments.

### Tissue microarray IHC scoring

**Figure 4:**
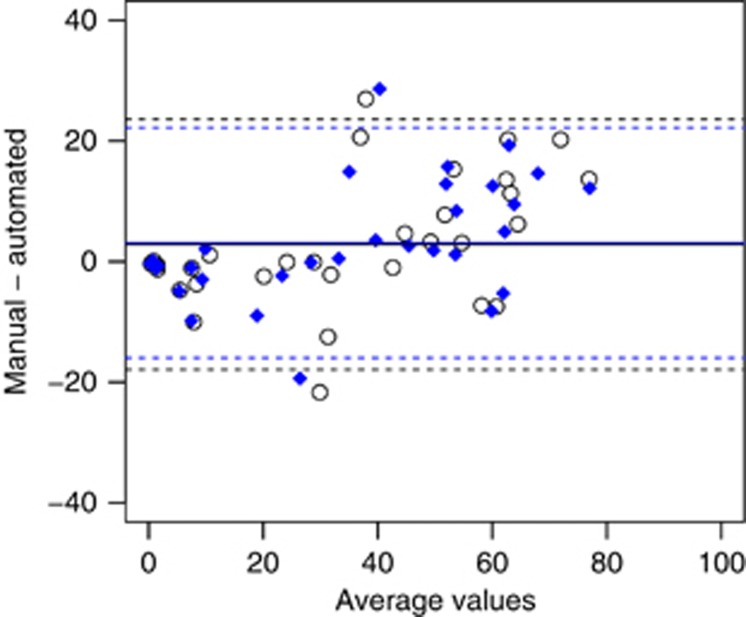
**Bland–Altman plot of percentage of positive cells identified in the Aperio software.** TMA spots are shown by black dots (pathologist A) and blue diamonds (pathologist B).

Intensity scores and percentage of positive cells were measured by the Aperio IHC Nuclear algorithm (Aperio Technologies) when provided with segmented tumour regions. Percentage of positive ER cells computed by Aperio were also evaluated (Figure 4). Agreements between scores calculated in Aperio were reported separately for intensity, and Allred and Quickscore proportion scores in terms of a two-rater weighted Kappa-squared statistic, 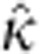 (Cohen, 1968) (Table 3). Inter-pathologist agreement was 0.96 for intensity scores, and 0.97 and 0.99 for proportion scores for Allred and Quickscore, respectively. In comparison, automated segmentations on average produced agreements of 0.89 for intensity scores, and 0.85 (Allred) and 0.87 (Quickscores) for proportion scores.

**Figure 5:**
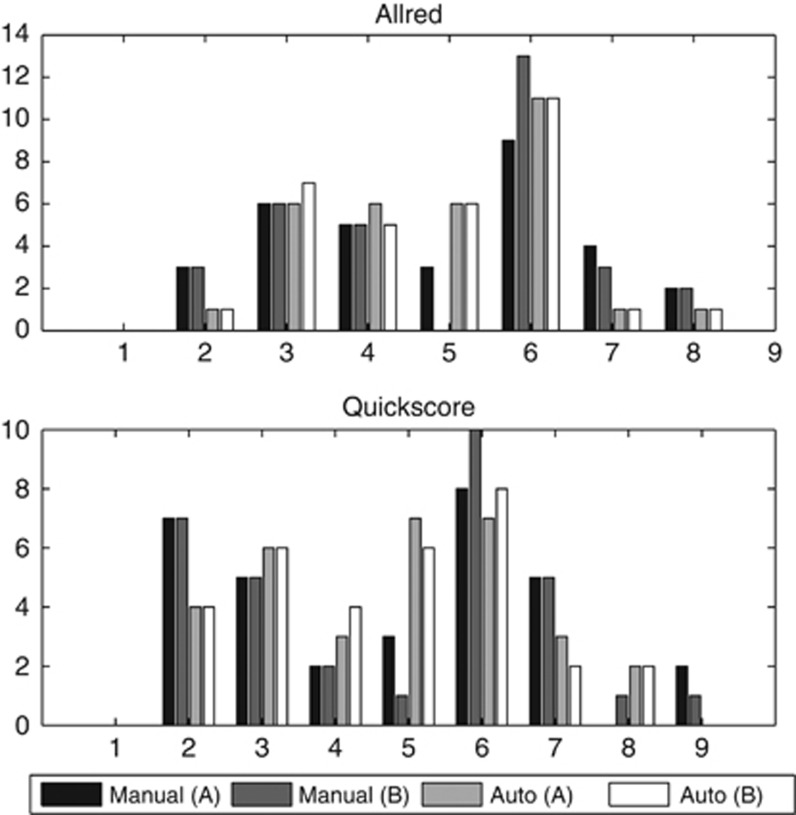
**Histogram plots of Allred scores and Quickscores extracted from manual and automated segmentations.**

Agreement for total Allred scores and Quickscores were computed by summing intensity and proportion scores. Comparisons between automated and manual segmentation masks resulted in average agreements of 0.91 (Allred) and 0.92 (Quickscore) (Table 4; Figure 5).

## Discussion

This study addressed the need for automated interrogation of TMAs using ER nuclear staining of primary breast cancer as an exemplar. Although previous studies have shown that image analysis can increase workflow and reduce inter- and intra-observer variability (Gurcan *et al*, 2009; Veta *et al*, 2014; Rohde *et al*, 2014), here we assess new image analysis software which shows potential to locate malignant tumour automatically with little intervention from human experts. Specifically in the reported study, automated segmentation was evaluated for the purpose of IHC scoring for a key nuclear stain, ER. Intensity, proportion and total (i.e. sum of intensity and proportion) IHC scores were reported, including inter-rater variability between pathologists with substantial Quality Assurance experience.

In the computer vision literature, evaluation of automated tumour segmentation algorithms is typically performed on a pixel-by-pixel basis. Our image analysis algorithm resulted in strong pixel-level agreements averaging *κ*=0.811 with two expert pathologists, falling only a little short of pathologist agreement (*κ*=0.908). However determining the impact of pixel-level disagreements in clinical practice is challenging. Therefore in this study, a method of categorising disagreements was presented, given the intended usage of the application is IHC scoring. Proportion of disagreement types (Table 2) revealed over 27% of pixel disagreements between automated and manual segmentations correspond to minor misalignment of tumour boundaries (Type 1) and therefore are inconsequential for IHC scoring as an epithelial cell cannot fit within these regions. Remaining disagreements (Type 2 and 3) varied considerably between TMA spots as shown by high s.d. and in keeping with the range of tumour and peri-tumoural stromal morphology seen in breast cancer.

When analysing intensity scores retrieved from manual and automated segmentations there was strong agreement (Table 3) with little difference when trained on scoring by pathologist A (0.92) compared with pathologist B (0.87) suggesting the automated approach is not heavily dependent on the individual pathologist. In contrast, agreements for proportion scores using automated segmentation masks were lower than reported agreement (inter-rater 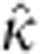
=0.97) with an underestimate in TMAs where there were high proportions of positive cells (Figure 4).

In the present study, IHC scores for ER were presented for two widely used scoring systems, Allred and Quickscore. IHC scores computed from automated segmentations were in strong agreement with scores from manually obtained segmentations (Table 4). Although reported values are not as high as inter-rater agreement, Allred scores from automated segmentations differed by at most one point. With further guidance and training examples, automation could be improved further, with the potential to replace manual annotations thereby increasing workflow. In addition, ER scores computed from automated segmentations were more consistent than scores retrieved from manual segmentations. In our experiments, Quickscores were identical for automated segmentation in all 32 TMA spots regardless of whom the system was trained on; similarly Allred scores were close and any differences were unlikely to have led to undertreatment even if used in a clinical setting. Using a more challenging nuclear biomarker, Ki67 positive cells (Mohammed *et al*, 2012) showed automation can provide standardized measures thereby reducing inter- and intra-observer variability. Furthermore, the image analysis algorithm used in this study is also applicable to other clinical measurements such as Histoscore (Jonat *et al*, 1986).

The dichotomy of tumour into ER+ve and ER−ve is essential for treatment decisions for endocrine therapy in clinical practice. In the present study, the Allred cut-off mark (>2), equivalent to USCAP 1% cut-off, resulted in almost complete agreement between all reported segmentations. Using Quickscores (cut-off >3), two TMA spots were labelled as ER+ve from automated segmentations and the same spots labelled ER−ve from manually obtained segmentations. The remaining 30 spots, 20 ER+ve and 10 ER−ve, were in complete agreement across all segmentations. Generally there was an overall agreement in treatment decisions, however results varied between scoring systems and non-standardized cut-offs. These discrepancies suggest more work may be required before automation is applied for treatment decisions as suggested for studies comparing visual and automated assessment of Ki67 markers in breast cancers (Fasanella *et al*, 2011; Mohammed *et al*, 2012). However, such numerically and proportionately small discrepancies may be insignificant in the setting of clinical trials with hundreds, and in some cases, thousands of participant patients. Given the need for TMA analyses, the present analysis of ER should be applicable to progesterone receptor (PR) or Ki67 staining, both of which are also intense nuclear stains. However, further issues, such as heterogeneity of PR protein expression, are also seen with markers not routinely used in clinical practice such as p53 (Coates *et al*, 2012). For Ki67 there remain multiple considerations to do with the antibody used, the methods of scoring and the cut-offs for positive v negative (Yerushalmi *et al*, 2010; Dowsett *et al*, 2011).

Despite pixel-level disagreements averaging 16% (Table 1), computed IHC scores from automated segmentation were in strong agreement with scores computed from manual segmentations (Allred; 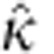
=0.91, Quickscore; 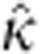
=0.92). With a larger number of tumour samples, it is likely that the application of this automated annotation approach will conclude similar outcomes to the more labour intensive manual annotations. Thus, the benefits of automation extend beyond the reproducibility of IHC scores to include changing the focus of research pathologists' workloads and the reproducibility of IHC scores. In principle, the generalisable automated method for tumour segmentation described here should be extendable to cytoplasmic or tumour cell membrane staining (e.g. HER2) and will be investigated in future work.

The automated segmentation method sometimes had difficulty distinguishing ER−ve cancer cells from ER−ve healthy epithelial cells. Availability of a greater number of TMA spots containing both ER−ve cancer cells and ER−ve healthy epithelial cells, along with accurate annotations of those spots for training, is therefore likely to be of substantial benefit. Indeed, increased volumes of annotated spots for training to more fully represent a range of staining conditions, tissue structures and artefacts can potentially improve segmentation accuracy and further align automated analyses with those of specialist pathologists. The impact of using larger volumes of annotations during training can be usefully explored in future work.

In summary, the use of automated annotations for scoring breast TMAs using the methods developed for and exemplified in this study concord closely with expert pathology reviews. 27% of pixel disagreements relate to minor misalignment of drawn tumour boundaries. Classification differences rarely resulted in a change of overall score that would be likely to change clinical management. Using the exemplar of nuclear ER staining, the methods of automated annotation employed here hold promise for reducing the expert pathology time required and speeding up analysis of IHC-stained TMAs from large data sets drawn from clinical trials.

## Figures and Tables

**Table 1:** Normalised contingency tables comparing segmentation labels in masks produced manually (Manual) by pathologist A and pathologist B and automatically (Auto)

	**Manual (A)**	
	**T**	**N**
**Manual (B)**		
*T*	0.270	0.049
*N*	0.043	0.638
**Auto (A)**		
*T*	0.221	0.097
*N*	0.092	0.591

	**Manual (B)**	
**Auto (B)**		
*T*	0.216	0.095
*N*	0.097	0.593

**Table 2:** Proportions of pixel label disagreements in each of the three types. The s.d. over spots is given in parentheses

**Comparison**	**Type 1**	**Type 2**	**Type 3**
Manual (A), manual (B)	0.227 (±0.144)	0.593 (±0.218)	0.180 (±0.227)
Auto (A), manual (A)	0.291 (±0.097)	0.604 (±0.161)	0.107 (±0.117)
Auto (B), manual (B)	0.305 (±0.119)	0.572 (±0.202)	0.123 (±0.122)

**Table 3:** Weighted Kappa-squared agreements for intensity and proportion scores computed from measurements obtained from the Aperio IHC algorithm

	**Intensity**		**Proportion**			
			**Allred**		**Quickscore**	
	**Manual (A)**	**Manual (B)**	**Manual (A)**	**Manual (B)**	**Manual (A)**	**Manual (B)**
Auto (A)	0.92	0.87	0.85	0.86	0.87	0.87
Auto (B)	0.92	0.87	0.84	0.85	0.89	0.88
Manual (A)	–	0.96	–	0.97	–	0.99

**Table 4:** Weighted Kappa-squared agreements for calculated Allred scores and Quickscores

	**Allred**		**Quickscore**	
	**Manual (A)**	**Manual (B)**	**Manual (A)**	**Manual (B)**
Auto (A)	0.91	0.91	0.92	0.92
Auto (B)	0.91	0.91	0.93	0.92
Manual (A)	–	0.98	–	0.99
