# Supplementary material for: Comparing computer-generated and pathologist-generated tumour segmentations for immunohistochemical scoring of breast tissue microarrays
Source: Br J Cancer. 2015 Sep 8;113(7):1075–80. doi: 10.1038/bjc.2015.309 (PMC4651129; doi:10.1038/bjc.2015.309)
Supplement: Supplementary Material 1 [file bjc2015309x1.pdf]

Supplementary Material

Computer-Generated Scoring Compared With Specialist Pathologist Scoring for Estrogen Receptor in Tissue Microarrays

Shazia Akbar[1], Lee B. Jordan[2], Colin A. Purdie[2], Alastair M. Thompson[3], and Stephen J. McKenna[1]

[1]School of Computing, University of Dundee, Dundee, DD1 4HN, UK

[2]NHS Tayside Department of Pathology, Ninewells Hospital, Dundee, DD1 9SY, UK

[3]Department of Surgical Oncology, MD Anderson Cancer Center, 1400 Pressler Street, Houston, Texas 77030, USA

1 Supplementary Materials and Methods

1.1 Rotation Invariant Superpixel Pyramid

An image analysis algorithm is proposed which automatically localises tumour by classifying superpixels (compact groups of pixels) as either tumour (T) or non-tumour (i.e. non-malignant) (N). A set of features is extracted from each superpixel and quantized using a learned codebook. Contextual information is captured at multiple scales in Rotation Invariant Superpixel Pyramids (RISP) to provide a rich representation of the tissue. A more detailed description of extracted superpixel features and the RISP representation follows.

1.1.1 Superpixel Features

A superpixel image representation is an over-segmentation in which similar pixels are grouped into perceptually consistent units. Superpixels are regularized to be compact and of similar size. We used Simple Linear Iterative Clustering (SLIC) by (Achanta *et al.* , 2012) to construct superpixels as it is computationally efficient and retains tissue structure (Figure 7). The compactness parameter in SLIC was set to 5. Fifty thousand superpixels were extracted per image such that the area of a single superpixel rarely exceeded that of a cell nucleus. Many nuclei were assigned two or more superpixels.

A set of features was extracted from each superpixel to describe its geometric and photometric properties as well as its adjacency relationship to neighbouring superpixels (Table 6). Features were normalized and concatenated to form a descriptor per superpixel. Extracted descriptors were then quantized, using a k-means codebook ($k = 200$).

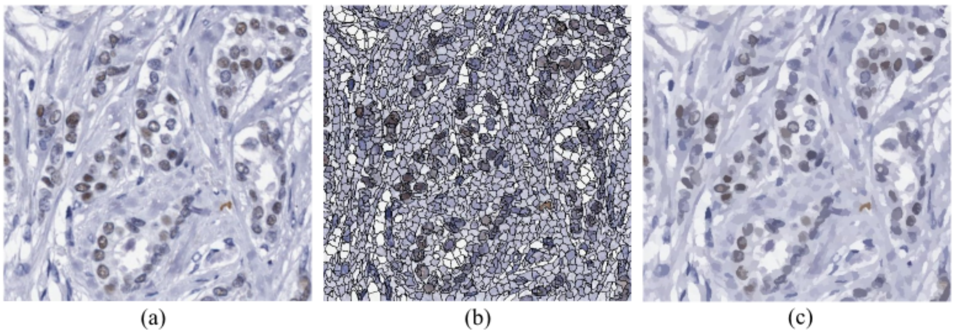

Figure 7: A TMA spot image (a) and corresponding SLIC superpixels outlined in black (b). Each superpixel is then rendered with the average RGB value of the pixels it contains (c).

Table 6: Features extracted from each superpixel.

Appearance	Mean red, green, blue, greyscale values
	Greyscale variance
	GLCM texture features (entropy, contrast, homogeneity, uniformity)
Geometric	Compactness
	Eccentricity
	Area
	Perimeter
Neighbors	Number of immediate neighbors
	Variance of superpixel perimeter shared between immediate neighbouring superpixels

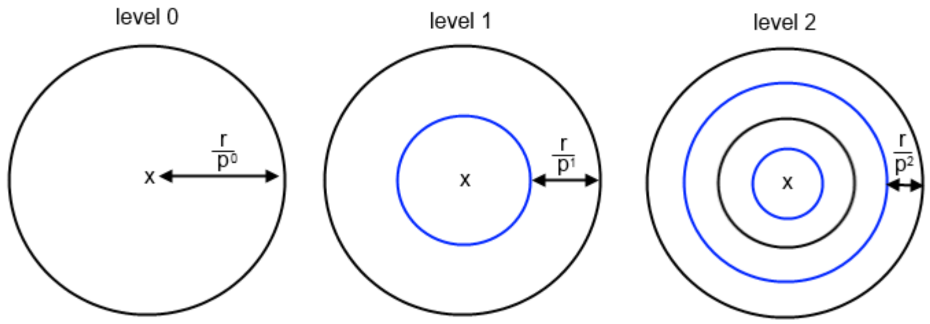

Figure 8: Level 0, 1 and 2 of the rotation invariant superpixel pyramid (RISP). Partitions are applied iteratively according to $p = 2$. Therefore for each ring in level l, two more are created in $l + 1$.

1.1.2 RISP Feature Representation

The Rotation Invariant Superpixel Pyramid (RISP) is an extension of the spatial pyramid proposed by (Lazebnik *et al.* , 2006) that captures context in a rotationally invariant manner (Akbar *et al.* , 2015). Rings were positioned in a circular window (radius 100 pixels) centred on a superpixel (Figure 8). The number of rings doubled with each level of the pyramid. For each ring, a histogram over the k superpixel codewords was extracted. At each level, these histograms were concatenated, resulting in a Spatial Bag-of-Superpixels (S-BoS) representation (Algorithm 1). These were computed at each of three pyramid levels and concatenated to form the multiscale RISP representation.

To provide complementary local information, RISP representations were concatenated with superpixel features for the central superpixel in the circular window. Resulting feature descriptors were used to train a linear support vector machine with Platt scaling (Platt, 1999) to output probability values between zero (N) and one (T) for each superpixel. To create binary segmentations, these probabilities were thresholded at 0.5.

Algorithm 1 Spatial Bag-of-Superpixels (S-BoS) histogram; one RISP level.

Image I, radius R, number of rings Q, codebook $\mathbf{C}$, number of superpixels, M

Run SLIC on I to generate superpixels, $\mathbf{s} = \{s_1...s_M\}$
Extract superpixel features, $\mathbf{F} = \{\mathbf{f}_1...\mathbf{f}_M\}$
for each superpixel, s_i, in **s do**
Identify superpixels **t** within circular window with radius, R, centered at s_i
Initialise spatial BoS histogram, H_i
for each superpixel, t_j, in **t do**
Lookup visual words, v_j for $\mathbf{f}_{t_j}$ in $\mathbf{C}$
Compute $d = ||c(t_j) - c(s_i)||$, c returns the center point of a superpixel
Increment $H_i = \left(v_j, \left\lfloor \frac{Qd}{R} \right\rfloor\right)$
end for
Normalise H_i
end for

1.2 Evaluation of Spot Segmentations

Tumour segmentation masks, S_A and S_B, were compared to produce two binary difference images, D_{A-B} and D_{B-A} (Figure 9). 8-connected morphological opening was then applied to D_{A-B} and D_{B-A}, resulting in O_{A-B} and O_{B-A}, respectively. The opening operation is an erosion following by a dilation with a circular structuring element with a radius of 20 pixels (Serra, 1982).

Type 1, Type 2 and Type 3 disagreements are defined as follows.

Type 1 Type 1 disagreements are pixels removed during the opening process i.e. pixels in D_{A-B} which did not appear in O_{A-B}. Similarly, the same comparison was performed between D_{B-A} and O_{B-A}. Pixels that differed before and after the opening operation were labelled Type 1.

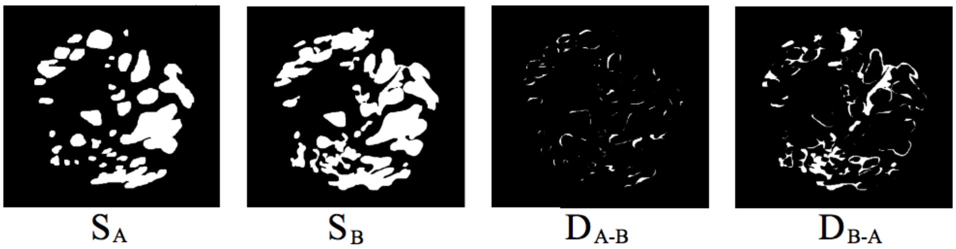

Figure 9: Binary tumour segmentation masks, S_A and S_B, hand-drawn by pathology experts and corresponding difference images, D_{A-B} and D_{B-A}. D_{A-B} and D_{B-A} denote disagreements between S_A and S_B.

Type 2 Region(s) in O_{A-B} and O_{B-A}, identified via 8-connected component analysis, which overlapped with agreed upon tumour region(s) were labelled Type 2. In the attached Matlab (.m) file, $FindConnectivity(O, S)$ returns regions in O which touch regions in S.

Type 3 Remaining regions, after identifying Type 1 and Type 2 disagreements, were termed Type 3.

References

Achanta R, Shaji A, Smith K, Lucchi A, Fua P, Süsstrunk S (2012) SLIC superpixels compared to state-of-the-art superpixel methods. Pattern Analysis and Machine Intelligence (PAMI) 34: 2274-2282.

Akbar S, Jordan L, Thompson AM, McKenna SJ (2015) Tumor Localization in Tissue Microarrays Using Rotation Invariant Superpixel Pyramids. International Symposium on Biomedical Imaging (ISBI), New York City, April 2015

Lazebnik S, Schmid C, Ponce J (2006) Beyond bags of features: Spatial pyramid matching for recognising natural scene categories. Computer Vision and Pattern Recognition (CVPR) 2: 2169-2178.

Platt J (1999) Probabilistic outputs for support vector machines and comparisons to regularized likelihood methods. Advances in large margin classifiers 10(3): 61-74.

Serra J (1982) Image Analysis and Mathematical Morphology. Academic Press, Inc. Orlando, FL, USA
