# Supplementary material for: Comparing computer-generated and pathologist-generated tumour segmentations for immunohistochemical scoring of breast tissue microarrays
Source: Br J Cancer. 2015 Sep 8;113(7):1075–80. doi: 10.1038/bjc.2015.309 (PMC4651129; doi:10.1038/bjc.2015.309)
Supplement: Supplementary Material 2 [file bjc2015309x2.pdf]

```matlab
%Journal: Breast Cancer Research and Treatment
%Title: Computer-Generated Scoring Compared With Specialist Pathologist Scoring for Estrogen Receptor in Tissue Microarrays
%Authors: Shazia Akbar, Lee B. Jordan, Colin A. Purdie, Alastair M. Thompson, Stephen J. McKenna
%Corresponding author: Stephen J. McKenna. email: S.J.Z.McKenna@dundee.ac.uk
%
%This Matlab code identifies Type 1 (T1), Type 2 (T2) and Type 3 (T3)
%disagreements between S_a and S_b which are both binary (i.e. logical)
%images. Disagreements are returned in binary format as well.
function [ T1, T2, T3 ] = FindDisagreementTypes( S_a, S_b )

D_fp = S_a & ~S_b;
D_fn = ~S_a & S_b;

%define structuring element
e = strel('disk', 10);
%perform morphological opening on D_fn and D_fp
O_fp = imopen(D_fp, e);
O_fn = imopen(D_fn, e);

T1 = (D_fp - O_fp) | (D_fn - O_fn);

T2 = FindConnectivity(O_fp, S_b) | FindConnectivity(O_fn, S_a);
T3 = (O_fp | O_fn) - T2;

end

%Find regions in O connected to regions in S
function [t2] = FindConnectivity(O, S)

    t2 = zeros(size(S));
    [~, L] = bwboundaries(O, 'noholes');   %perform CCA

    for idx = 1:max(max(L))
        %find outline of region by dilation
        outline = imdilate(L==idx, strel('disk',1));
        %if outline intersects with S then connected
        if(sum(S(outline)))
```

```
                t2(L==idx) = 1;
            end
        end

end
```
